# CYP1A expression in freshwater fish of western New York as an indicator of pollution levels

**DOI:** 10.1002/ece3.8526

**Published:** 2022-01-25

**Authors:** Rebecca Williams, Theresa Taggart, Kayla Ganger, Teri Koetsier, Seema Johnson, Amber Dinchman

**Affiliations:** ^1^ Houghton College Houghton New York USA; ^2^ College of Wooster Wooster Ohio USA

**Keywords:** adaptation, CYP1A, fish, freshwater, pollution, western New York

## Abstract

Various species of freshwater fish regulate the expression of certain proteins in response to environmental contamination. Previous research has shown that CYP1A expression increases in response to contaminant levels and can result in increased tumor formation. Fish in contaminated environments would thus benefit by downregulating the expression of CYP1A to reduce tumor prevalence as an adaptive strategy. Alternatively, monitoring of the CYP1A protein in fish can serve as a bioindicator of the pollution level of an environment. This study evaluated CYP1A expression in twelve different species of freshwater fish from seven bodies of water throughout western NY including Cuba Lake, Genesee River, Hanging Bog, Love Canal, Moss Lake, Rushford Lake, and Tifft Nature Preserve. Western blot analysis was used to measure CYP1A expression as a marker of site pollution and potential fish population adaptation. It was hypothesized that low CYP1A expression at a site with known contamination would suggest signs of adaptation to pollution levels present. Furthermore, if at least one sample from a species showed CYP1A expression, then the CYP1A antibody (Caymen Chemical, USA; 173132) had compatibility with that species, eliminating falsely suspected adaptation. The results from this study suggest possible adaptation of fish may be occurring in the polluted Tifft Nature Preserve and Genesee River. In contrast, CYP1A expression in fish from Cuba Lake, Hanging Bog, Love Canal, Moss Lake, and Rushford Lake appear to represent known pollution levels and adaptation is not likely occurring. Results from this study are preliminary and next steps include collection and analysis of sediment to provide a stronger correlation between pollution at sites and CYP1A expression.

## Graphical abstract

Biomarker CYP1A expression was measured and analyzed in twelve fish species across seven sites in western New York. We aimed to explore whether CYP1A analysis demonstrated an accurate reflection of site health or displayed characteristic adaptive efforts. Conclusions were made based on known contaminant data and known remediation efforts.

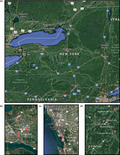

## 1 INTRODUCTION

Freshwater fish can reveal information concerning levels of aqueous contamination through regulation of the CYP1A protein. Fish that feed along the floor of water systems are more likely to indicate contamination than fish that feed along the surface, because toxins often reside in the sediment of freshwater bodies due to their low aqueous solubility (Andrade et al., 2004). Many contaminants can induce CYP1A expression, including both synthetic and natural sources such as microcystins (Garcia & Martinez, 2012). Polycyclic aromatic hydrocarbon (PAH) toxins in particular have been found to have extremely low aqueous solubility, on the order of nanograms per liter (Fetzer, 2002). Although these CYP1A‐inducing toxins are found in the sediment of freshwater, they may not always induce CYP1A expression in bottom‐feeding fish. In one study of the River Narva, researchers found that despite elevated PAH levels from nearby mining, CYP1A induction was low, suggesting adaptation of fish over time to high levels of toxins in their environments (Tuvikene et al., 1999).

This current study explored the effect of contaminants on CYP1A expression in freshwater fish from seven bodies of water across western New York as a measure of adaptation. Bottom feeding freshwater fish included in this study are black bullhead (*Ameiurus melas*), brown bullhead (*Ameiurus nebulosus)*, pumpkinseed (*Lepomis gibbosus*), largemouth bass (*Micropterus salmoides*), white suckers (*Catostomus commersonii*), rock bass (*Ambloplites rupestris*), golden shiners (*Notemigonus crysoleucas*), and Rudd (*Scardinius erythrophthalmus*). Fish that feed along the surface or upper levels of water bodies were included as other possible bioindicators. These included bluegill (*Lepomis macrochirus*), Northern pike (*Esox lucius*), and fallfish (*Semotilus corporalis*).

The migratory and feeding patterns of each species impact which contaminants they are exposed to. Largemouth bass also feed along the surface or shallows of the water. In the summer, they tend to move toward deeper waters after spawning season (Minnesota DNR). Northern pike swim closer to the surface on sunny days and migrate during their spawning season between February and March (Ovidio & Philippart, 2003). White suckers migrate anywhere from 100 yards to 4 miles during their spawning season, from April to June (Raney & Webster, 1942). Bluegill tend to inhabit vegetated, quiet, shallow areas and do not migrate. Occasionally, they swim in deeper areas to avoid higher water temperatures (Schultz, 2004). Pumpkinseed do not migrate and feed primarily on gastropods on both surface and bottom layers of the water (Mittelbach et al., 1999). Golden shiners never migrate and feed in numerous water levels (Reebs, 2002). Rock bass live in small, shallow, cool streams among the rocks or vegetation (Schultz, 2004). Fallfish feed on plankton and insects in clear areas of water and tend to stay in their native areas (NYS Department of Environmental Conservation). Rudd feed along the surface, middle, and bottom of water and remain with the same school of fish throughout their lifetimes (Schultz, 2004). Lastly, Brown bullhead (*Ameiurus nebulosus*) are especially good indicators of water and sediment contamination. Their benthic nature causes them to take up and bioaccumulate toxins from the sediment (Azevedo et al., 2012). Furthermore, bullhead have the ability to live in highly contaminated environments and have demonstrated adaptive responses to CYP1A inducers by increasing tumor suppressor p53 expression and decreasing CYP1A expression (Williams & Hubberstey, 2014).

On the other hand, direct correlations between levels of CYP1A expression within the fish and pollution levels in the water validate procedures involving the measurement of this gene for the quantification of water pollution (Azevedo et al., 2012; Pelayo et al., 2011). Bullhead in locations with tremendous amounts of pollutants such as PAHs and polychlorinated biphenyls (PCBs), another class of contaminants that are prevalent in sediment, have demonstrated increased CYP1A expression in addition to skin and liver tumors (Pyron et al., 2001). PAHs are produced as a result of contamination from industrial effluents, agricultural runoff, municipal waste, and incomplete combustion of petroleum, gas, coal, and wood (Willett et al., 2001; Andrade et al., 2004). This type of contamination is the result of social and economic development, as PCBs and PAHs are often dumped into bodies of water as industrial waste (Nacci et al., 1999). Members of this group of PAHs containing four or more benzene rings (e.g., benzo[a]pyrene, BaP) specifically interact with the CYP1A family of enzymes by binding to aryl‐hydrocarbon receptors (AHRs) resulting in elevated levels of CYP1A expression (Willett et al., 2001; Wills et al., 2010). The CYP1A protein can then metabolize PAHs such as BaP into mutagenic end forms that interact negatively with DNA, resulting in the formation of adducts (Hodek et al., 2013).

Upregulation of CYP1A is a tightly monitored system used to eradicate chemicals from the body (Ma & Lu, 2007). The role of CYP1A in the removal of toxins makes the enzyme useful in contaminated environments. Activated CYP1A adds an oxygen atom to a carcinogen, allowing the toxin to become soluble in water, followed by excretion from the body (Pfeifer et al., 2002). However, CYP1A can also metabolize those toxins to their mutagenic form, leading to tumor formation and other complications within the fish (Wills et al., 2010). Decreased CYP1A expression reduces the possibility of these negative effects and consequently increases the likelihood that a fish will survive in a contaminated environment.

Research suggests that fish with a higher tolerance (i.e., downregulation of CYP1A to reduce tumor formation) for contamination will be more fit to survive and produce offspring that also possess higher tolerance for similar environments. Over time, this causes a genetic shift toward a population that is resistant to pollution (Klerks & Levinton, 1989; Nacci et al., 1999; Wills et al., 2010). Alternatively, if a trait is not inherited and occurs within the lifetime of an organism, this is considered to be a nongenetic inheritance (Meyer et al., 2002). Adaptation may not occur at all in small freshwater systems if the population goes extinct before resistance can develop or the group is not genetically diverse enough to allow for adaptation (Hamilton et al., 2017).

Studies have confirmed that resistance to contaminants can be heritable rather than nongenetically maternally influenced. A study by Wills and colleagues demonstrated that killifish can adapt to carcinogens like BaP. Killifish larvae were bred in a laboratory using parent fish from the Elizabeth River (ER) and King's Creek (KC) in Virginia. The larvae were then exposed to aqueous BaP or DMSO (Wills et al., 2010). The Elizabeth River is known to be contaminated with creosote, a complex mixture of PAHs (Bieri et al., 1986). Results showed that laboratory‐raised KC larvae had significantly higher CYP1A activity after exposure to BaP compared to ER larvae. The authors demonstrated that ER fish appeared to have acquired a heritable defense mechanism against genotoxicity and subsequent carcinogenesis (Wills et al., 2010).

More recently, using a model of C. elegans expressing zebrafish CYP1A, Harris and colleagues showed that CYP1A protected against BaP exposure and was slightly less protective against a mixture of PAHs from a contaminated site. The mechanisms of protection against specific contaminants have yet to be elucidated (Harris et al., 2020). Interestingly, Wincent and colleagues tested 15 oxygenated PAHs in HaCaT cells and found that 9 of them induced CYP1A1 gene expression. However, only 5 of these induced EROD activity. They also found that 11 of the oxy‐PAHs inhibited recombinant human CYP1A activity. Therefore, some oxy‐PAHs can be activators of AHR signaling, but inhibitors of CYP1 function (Wincent et al., 2016). CYP1A expression in response to contamination in many model systems is a complex topic. Specifically, the relationship between evolutions of CYP1A expression in fish during contaminant exposure deserves further attention. Mixtures of contaminants can also be challenging to evaluate, and this should be addressed in the future.

**FIGURE 1:**
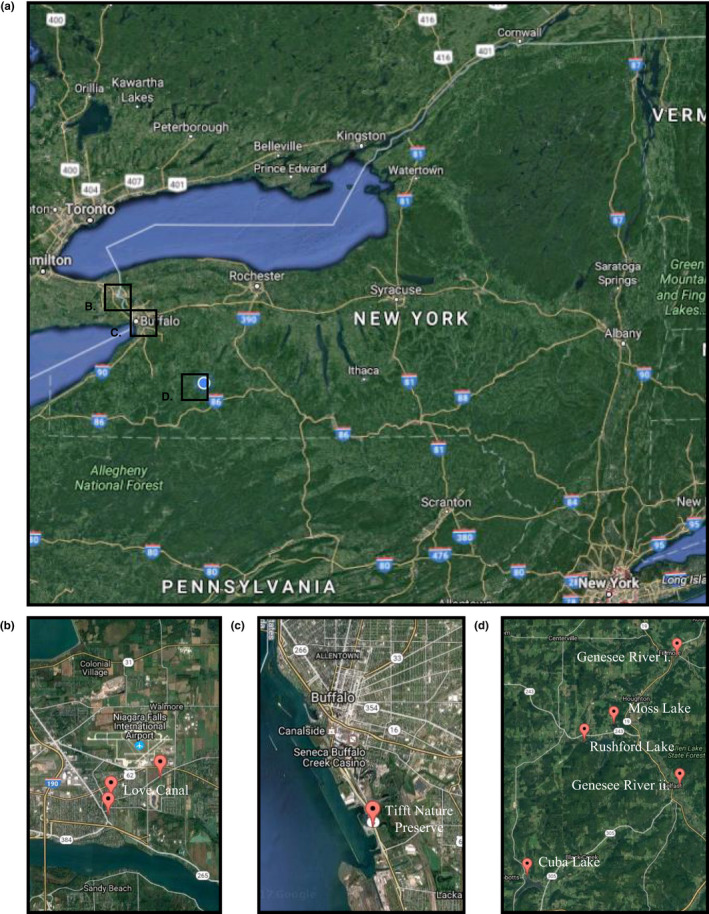
Sampling Sites Across Western New York. This map shows locations where 12 different species of fish were captured. Part (a) depicts the geographic range of sites and parts (b), (c), and (d) are enlarged to provide greater detail and demonstrate proximity between sites. Specific geographic coordinates (latitude, longitude) and species of fish caught are listed in Table 2

The purpose of this study was to screen CYP1A protein expression in species of freshwater fish from seven bodies of water across western NY: Cuba Lake, Genesee River, Hanging Bog, Love Canal, Moss Lake, Rushford Lake, and Tifft Nature Preserve (Figure 1). Screening CYP1A expression can serve as a method of evaluating site health and contamination. It was hypothesized that if low CYP1A levels were observed in a site with known contamination (Table 1), then fish populations may be adapting for the purpose of survival. Furthermore, if CYP1A expression was observed in at least one sample of a species, then the CYP1A antibody used was compatible with that species and would eliminate false assumptions of adaptation.

Some of the sites included in this study had various known contaminants present while others did not have any known sources of contamination. See Table 1 for details.

**TABLE 1:** Contaminants present at each site

Site Name	Sources of Contamination
Moss Lake (ML) Figure 1d	Relatively clean body of water, owned by Nature Conservancy (Underhill, 1999)
Tifft Nature Preserve (T) Figure 1c	Transshipment center for coal and iron ore, landfill from 1950s−1960s. (Tifft Nature Preserve, 2016) Superfund site (inactive hazardous waste site). Barrels of naphthalene were uncovered and removed in 1983. (Spiering, 2009) Soil content varies throughout the preserve. Former soil chemistry analysis reported the presence of minerals (calcium, magnesium, potassium and phosphorus) throughout the preserve as a result of dumping of slag. Surface soil samples identified heavy metals (chromium, copper, lead, zinc) throughout the preserve as a result of dumping of foundry sand and slag. Deep, underlying soil contents include coal, cinder, slag, construction and foundry waste. (Klips et al., 1993)
Rushford Lake (RL) Figure 1d	Oil wells in nearby Rushford and Cuba, NY. (Rhodes, 2016)
Love Canal (LC) Figure 1b	Municipal, industrial hazardous waste dump site in the 1920s. Carcinogens, including benzene, corroded waste drums. Superfund site. (NY DEC)
Genesee River (GR) Figure 1d	Urban and industrial runoff near Rochester, NY (NY DEC)
Cuba Lake (CL) Figure 1d	Seneca Oil Spring (Minard, 2016)
Hanging Bog (HB) Figure 1d	No known contaminants. Man‐made bog, current wildlife management area. (NY DEC)

## 2 MATERIALS AND METHODS

### 2.1 Fish capture

Fish for this study were captured from seven sites across western New York (Figure 1 for map, Table 2 for details) using hoop nets during the months of March–November 2016 (New York State Department of Environmental Conservation License to Collect or Possess: Scientific #2029, institutional animal care and use committee permissions were granted from Houghton College #16‐01). Nets were left at site locations overnight and collected the next day. Not all fish captured were used but at least *n* = 1 of a species was used if caught. Weight and length of species were noted but sex and age were not. Sampling sites were visited more than once during this time period in an effort to maximize collection data. When sampling efforts and varied techniques resulted in repeated attempts without a change in the fish collected or n of species, data analysis was pursued with the population collected, regardless of species type and number, in an effort to give best representation of site health in a specified time frame. Sampling did not continue during the next field season (2017) as this seemed contradictory to giving an accurate reflection of the health of the aquatic site by sampling over the course of several years, and this would introduce additional unreliable factors, including mixed fish populations over time where remediation efforts were taking place (e.g., Tifft Nature Preserve, Genesee River).

**TABLE 2:** Site locations and fish species captured across western New York

Location (latitude, longitude)	Fish Type (quantity)
Love Canal 43.095662, −78.934339 43.0823488, −78.9596629	Golden Shiner (2) 1.1, 1.3 Northern Pike (1), 1.2 Fallfish (1), 2.1
43.088226, −78.958560	Northern Pike (2), 3.1–3.2 Rudd (2), 4.1–4.2 White suckers (4), 5.1–5.4
Rushford Lake 42.383232, −78.222755	Brown bullhead (4), 1.1–1.4
Moss Lake 42.399619, −78.185771	Black bullhead (10), 1.1–1.10 Brown bullhead (6), 2.1–2.6
Genesee River 42.463088, −78.106179	Rock bass (3), 1.1–1.3
42.341876, −78.102836	Rock bass (2), 1.4–1.5
Tifft Nature Preserve 42.8461723, −78.8616481	Bluegill (10), 1.1–10.1 Largemouth bass (1), B1 Brown bullhead (1), A1
Cuba Lake 42.257847, −78.294773	Rock bass (3), 1.1, 2.1–2.2 Pumpkin seed (6), 3.1–3.6
Hanging Bog 42.307603, −78.248694	Brown bullhead (1), 1.1 Bluegill (1), 2.1 Rock bass (1), 2.2

### 2.2 Tissue extraction, Western blot, and densitometric analysis

Samples were brought back to the lab and sacrificed using MS‐222 (Sigma, USA). Animal protocols were in accordance with Houghton College's Animal Care Committee. All animals were treated and euthanized humanely. Samples were stored in RNA later (Sigma, USA) at room temperature overnight before protein extraction and then moved to a −20°C freezer for long‐term storage. CYP1A protein levels can be measured in various organs, particularly the liver, where they are most concentrated. This makes it a primary organ of study for measuring contamination levels (Van Veld et al., 1997).

Protein was extracted (500 μl of Tris‐HCl pH 7.4, 100 μl of IGEPAL, 0.25% NaDeoxycholate, 1 mM EGTA pH 8.0, 0.2% SDS, 150 mM NaCl, 1.5% antifoam A) from each of the liver tissues that had been preserved in RNAlater for analysis on 10% acrylamide (Bio‐Rad, USA) (Cynthia et al., 1997). Samples were analyzed using Bradford assay and 35 μg samples were prepared. A positive control (BR2) was loaded onto each gel. This sample is a first‐generation farm‐raised bullhead whose parents were taken from a moderately clean site (Belle River, Ontario, Canada) and exposed to 75 mg/kg of BaP to induce CYP1A expression.

Membranes were blocked in 1% milk for one hour. The actin (Millipore, USA; MAB 1501), CYP1A (Caymen Chemical, USA; 173132), and goat anti‐mouse hrp antibodies (Sigma, USA; A4416) were applied at a concentration of 1:10,000. Actin was used as a loading control for each blot. All blots were imaged for less than 2 min using West Femto SuperSignal substrate (Thermo Fisher, USA).

### 2.3 Densitometric analysis

Band density was measured using Image Lab software and quantified in terms of relative front of bands and volume of each band (Bio‐Rad, USA). The band volumes were used in densitometric analysis. Due to variation in actin intensity, each sample's CYP1A volume was divided by the volume of actin it expressed in order to normalize the dataset on a blot. This yielded a CYP1A:actin ratio for each sample. The BR2 control was expressed consistently across these blots but its intensity also varied. The average CYP1A:actin expression for all samples on a blot was normalized for this variation by dividing each CYP1A:actin ratio by the quantified BR2:actin ratio on that blot.

### 2.4 Statistical analysis

We tested the hypothesis that CYP1A expression would not differ between sites that varied in pollution levels using a one‐way ANOVA in Microsoft Excel. Statistical significance was determined by using a one‐way ANOVA to compare CYP1A expression between all sites (*p* < .05).

### 2.5 Sampling site data

All known sources of pollution and contamination (former and up to time of sampling) were collected from either available literature sources or known persons with affiliation of sites. Search for this information was conducted between January 2016 and November 2021 and is presented in Table 1. No change in information was found between the end of sampling (November 2016) and present. Sediment samples were not collected at time of sampling and are not relevant because of mixed fish populations analyzed from the majority of our sites, some of which consist of fish that either exhibit either migratory and nonmigratory patterns, or reside in different levels of the water column, or both. For these reasons, fish collected may not accurately correlate with point sediment samples taken.

## 3 RESULTS

### 3.1 CYP1A expression in western New York

The aim of this study was to quantify pollution levels of the following bodies of water across western New York: Cuba Lake, Genesee River, Hanging Bog, Moss Lake, Love Canal, and Tifft Nature Preserve (see Figure 1 for map and Table 2 for site details). To do this, CYP1A expression was measured in the liver tissue of twelve different species from various sites (see Table 2).

CYP1A expression in each liver tissue sample was measured using Western blot analysis. Densitometric analysis was used to quantify and rank CYP1A expression. Densitometric values considered CYP1A expression of all fish species for a particular site. Sites were compared to determine relative known pollution levels between them. In the order of highest to lowest magnitude of average CYP1A expression, the following results were obtained: Love Canal, Cuba Lake, Rushford Lake, Tifft Nature Preserve, and Moss Lake. No CYP expression was detected in both the Genesee River and Hanging Bog (densitometric value = 0). The Love Canal had 48.3× more CYP expression than Cuba Lake, whose fish had 1.08× more CYP expression than Rushford Lake, whose fish had 22.6× more CYP expression than Tifft Nature Preserve, whose fish had 21.5× more CYP expression than Moss Lake.

Results of the one‐way ANOVA demonstrated that site did not have a significant effect on CYP1A expression (*p* = .637). Though no significant difference in CYP1A expression between sites was observed, varying levels of CYP1A were detected between sites.

### 3.2 CYP1A antibody species recognition

From the twelve different species that were captured, the following showed cross reactivity with the CYP1A antibody (Caymen Chemical, USA; 173132) that was used: black bullhead, bluegill, brown bullhead, fall fish, golden shiner northern pike, rudd, and white sucker. No cross‐reactivity was observed with the following species: rock bass and largemouth bass.

## 4 DISCUSSION

In this study, CYP1A expression was measured in 12 different species of fish from 7 different sites across western New York (see Figure 1). The results of this experiment show that fish from the Love Canal appeared to have the greatest CYP1A expression among the fish from all locations, despite the inclusion of two fish species that feed along the upper surface of the water column (Table 2). The Love Canal tragedy is well documented and has a history of pollution. This partly dug canal, once envisioned to be a source of hydroelectricity, was never completed and was instead used as a dump site by the Hooker Plastic and Chemical Company between 1942 and 1953 (Phillips et al., 2007) who then covered the waste with dirt (Beck, 1979). After a record rainfall, leaching began and chemicals were distributed throughout the area, resulting in unusually high miscarriages and birth defects (Beck, 1979). Subsequently, the site was declared an environmental state of emergency twice and residents were evacuated. As a result of clean‐up and remediation, this Superfund site is now habitable (EPA). Similar to what was observed in fish from the Love Canal, the effects of PAHs in the sediment were previously observed in bullhead in the form of elevated fluorescent aromatic compounds, EROD activity, CYP content and CYP1A protein when compared to Black Creek, a reference site (Eufemia et al., 1997).

The Hanging Bog and Moss Lake have undetectable and low CYP1A expression, respectively, which corresponds to the known lack of aryl hydrocarbon antagonists at both of these sites (Table 2), suggesting that findings obtained from both likely provide an accurate representation of the current quality of water. Moss Lake, in particular, could be considered a reference site used for comparison. The brown bullhead from Hanging Bog, Moss Lake, Rushford Lake, and Tifft Nature Preserve has shown success with Caymen Chemical, USA; 173132 in a previous study, eliminating the possibility of false conclusions (Williams & Hubberstey, 2014). For the Hanging Bog, most fish species showed cross‐reactivity to Caymen Chemical 173132 previously or at other sites (brown bullhead, bluegill) and so false adaptation can likely be eliminated, and this site can likely serve as a reference site as well.

**FIGURE 2:**
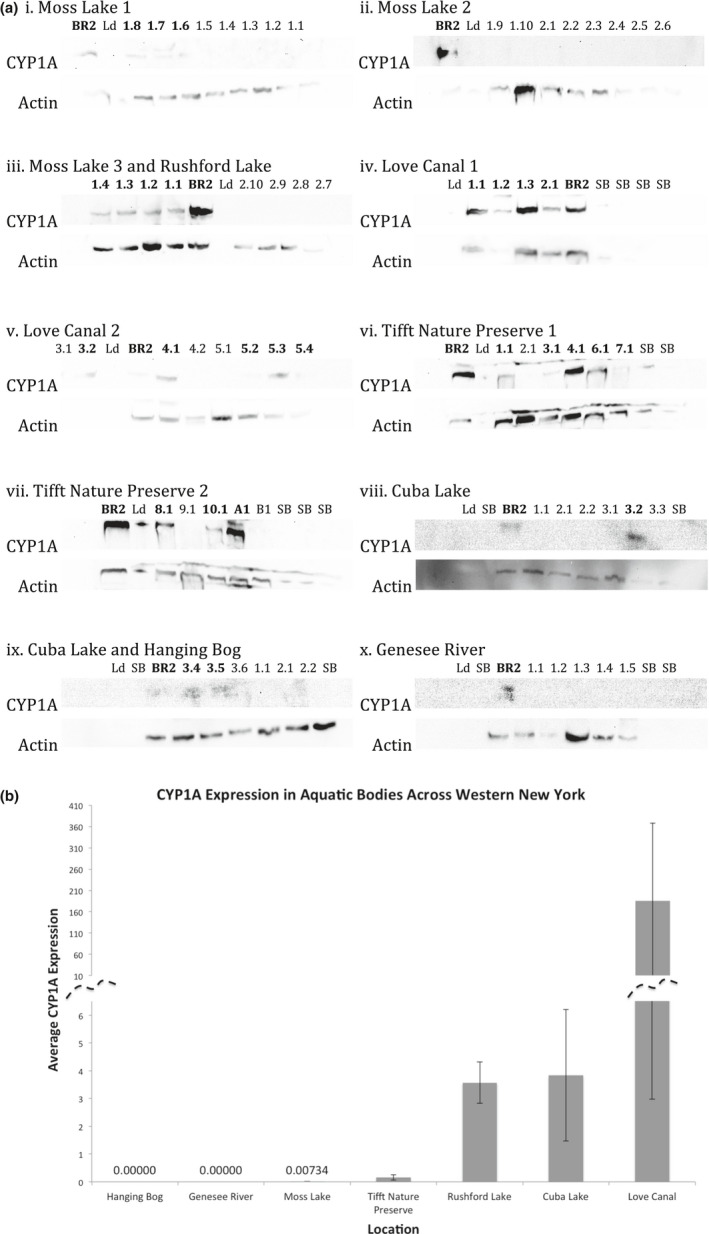
CYP1A and Actin Expression in Freshwater Fish of Western New York. (a) Spaces labeled “SB” were filled with sample buffer in all Western blots and should not express CYP1A or actin. Some SB wells do show expression, most likely due to spillover from neighboring wells. Spaces labeled “Ld” represent the protein ladder. Blot *iii* contained samples from Rushford Lake (1.4, 1.3, 1.2, and 1.1) and Moss Lake (2.10, 2.9, 2.8, 2.7). Blot *ix* contained samples from Cuba Lake (3.4, 3.5, and 3.6) and Hanging Bog (1.1, 2.1, 2.2). Each sample expressed quantifiable actin. Each blot showed CYP1A expression in the control BR2 sample. Samples in bold print showed CYP1A expression. All sample names can be matched to their species in Table 2. (b) CYP1A and actin expression were quantified using chemiluminescence. The amount of CYP1A of a sample was divided by the amount of actin for that sample, yielding its CYP1A:Actin ratio. The average CYP1A:Actin expression for all samples on a blot was normalized for variation by the quantified BR2:actin ratio on the same blot. The error bars represent +/‐ SE of the mean of samples for each site. Finally, a one‐way ANOVA was conducted for the dataset (*p* = .637), meaning no significant difference in CYP1A expression between sites was observed

In comparison to Moss Lake and the Hanging Bog, which are known to be relatively clean bodies of water, both the Genesee River and Tifft Nature Preserve have known sources of contamination (Table 2). Based on this information, the CYP1A expression observed was lower than expected and suggests adaptation could be occurring in these fish. Alternatively, the results observed in Tifft Nature Preserve could be the outcome of ongoing remediation efforts of the Buffalo Museum of Science (Tifft Nature Preserve, 2016), reducing potential CYP1A inducers and subsequently, CYP1A expression. Beyond remediation, most of the fish captured at Tifft Nature Preserve were bluegill, which are known to feed along the surface or upper levels of water (Table 1). Therefore, they may not be exposed to the same contaminants as bottom feeders and thus may not accurately reflect pollution levels or potential adaptation (Andrade et al., 2004). All samples taken from the Genesee River were rock bass. This species showed no cross‐reactivity with Caymen Chemical, USA; 173132 in any other samples, providing no evidence against a false conclusion for adaptation in the Genesee River (Figure 2a), which is possible in rock bass (Brammell et al., 2004). Alternatively, results obtained from the Genesee River may accurately reflect ongoing remediation efforts similar to Tifft Nature Preserve (NYDEC).

Next, Rushford Lake and Cuba Lake had intermediate levels of CYP1A expression in comparison to the Love Canal (lower than) and Moss Lake and Tifft Nature Preserve (higher than). Both of these bodies of water are known to be contaminated by sources of oil, possibly resulting in similar CYP1A expression levels in fish from both of these sites. PAHs found in oil are able to induce CYP1A expression as an indicator of exposure to petroleum and oil spills can result in persistent CYP1A expression as was evidenced up to 10 years after the initial *Exxon Valdez* oil spill in two nearshore species of fish in Prince William Shores, Alaska. Recent exposure could be more accurately determined by measuring FAC in bile (Jewett et al., 2002). In another study, caged salmon, purposed for a sentinel response, showed a time and thus dose‐dependent response to PAHs after the *Braer* oil spill, demonstrating the relationship between the presence of oil and CYP1A expression (Stagg et al., 2000). All fish caught at these two locations are bottom feeders (brown bullhead, pumpkinseed, and rock bass) who do not migrate, dissuading the idea that they do not accurately reflect their environment. These three species have served as bioindicators (Baumann et al., 1991; Facey et al., 2005; van der Hurk et al., 2017) and demonstrated the ability to adapt as a species (Brammell et al., 2004; Murdoch & Hebert, 1994; Vila‐Gispert et al., 2007) suggesting that unless adaptation is occurring in these populations, CYP1A expression observed is an accurate representation of the contamination present in the sediment and these fish can be used for bioindication.

Though variation in CYP1A expression was observed across sites, no significant difference was measured (*p* > .637). We propose that this could have been due to diversity in the fish populations used. Only Moss Lake, Rushford, and the Genesee River had homogenous populations of fish. Cuba Lake, Tifft Nature Preserve, Love Canal, and the Hanging Bog had heterogenous populations and this factor needs to be taken into consideration when drawing conclusions of fish used as bioindicators or suggesting adaptation of a population (Zhang et al., 2017). Because the Hanging Bog is considered to be a clean site, no CYP1A expression was expected. However, Cuba Lake, Tifft Nature Preserve, and the Love Canal have known sources of contamination in addition to mixed fish populations (Table 2). One study by Rasanan and colleagues compares the CYP1A expression of whitefish to that of northern pike when exposed to two PAHs, retene, and pyrene. In nature, both species are exposed to retene when they lay eggs along the bottom of a lake. The study found that retene induced CYP1A expression in whitefish, while pyrene did not (*p* < .05) and was not measurably expressed in northern pike in response to retene (Rasanen et al., 2011). Variation in the response of different species to specific PAHs demonstrates that it is helpful to study homogenous fish populations so that conclusions could be drawn about individual species from different locations. The lack of statistical significance observed in this study is likely due to the variation between sites in both species and type of pollution. In addition to this variation, there was likely variation in age of the fish collected. This variation in age could contribute with the varied response in CYP1A expression. It has been shown that juvenile fish have increased sensitivity to toxicants due to continued development (Mohammed, 2013). In the future, collecting a consistent sample of the same species would improve accuracy.

Another aspect to consider is the interaction of various chemicals in the sediment and how those combinations may influence induction of CYP1A expression. In a study completed by Willet et al., killifish were dosed with either 5 mg/kg or 50 mg/kg of BaP or fluoranthene (FL), another PAH. BaP induced significant CYP1A‐mediated EROD treatment on its own but in conjunction with FL, did not. Furthermore, FL was able to noncompetitively inhibit BaP‐induced EROD activity, possibly by upstream mechanisms (Willett et al., 2001). On the other hand, a study by Wassenberg and Di Giulio showed that FL and BNF, another known PAH, together increased deformity in killifish embryos. In this study, embryos were exposed to a wide variety of PAHs individually and in combination with each other. Some of these included BNF, ANF, PBO, and FL. ANF is a known CYP1A inhibitor, as well as an AHR antagonist. BNF on its own induced significant levels of EROD activity, while ANF significantly inhibited EROD activity. But when BNF and ANF were combined, the embryos deformed to the extent that EROD activity could not be determined. The same was true when BaP was combined with ANF. BaP did not cause significant EROD induction on its own, but combined with ANF, induced significant deformity in embryos. Similarly, FL did not affect the embryos EROD levels on its own, but when combined with BNF, high levels of EROD induction were observed. PBO elevated levels of deformity in embryos on its own and when combined with BNF, significant EROD activity was induced (Wassenberg & Di Giulio, 2004). Observations from this study suggest a need to consider the effects of multiple compounds on CYP1A expression.

Recent research shows that the relationship between CYP1A and PAH exposure can be protective in some cases (Harris et al., 2020). Oxygenated PAHs can also induce AHR signaling but inhibit CYP1 activity in HaCaT cells (Wincent et al., 2016). CYP1A expression in response to PAH exposure is a complex topic. Certainly, eco evolutionary interactions can also be complex, and future studies will aim to clarify these relationships. For example, outcomes where evolution is suspected to balance other effects are at risk of being overlooked. The idea of rapid evolution within freshwater systems involves many factors. Hypotheses about reciprocal interactions between ecology and evolution should be considered in relation to this study and future investigation of this topic (Kinnison et al., 2015).

## 5 CONCLUSION

CYP1A expression has been used in the past as a bioindicator of pollution. However, some fish may adapt to pollution and contamination over time so that they eventually express much lower levels of CYP1A protein (Chivittz et al., 2016; Wills et al., 2010). This study is the first of its kind in western New York and should be continued so that CYP1A expression can be used to monitor pollution levels in various bodies of water. If contamination is decreasing due to remediation efforts, fish may express lower levels of CYP1A. But if fish are expressing lower levels of CYP1A without any change in pollution, there is a strong possibility the suspect population has adapted over time to its surroundings.

For future studies, sediment in all sites should be monitored frequently for fluctuations in contaminants over time and distribution to determine whether CYP1A inducers are present and leading a population toward adaptation. Sediment composition would also help to determine which compounds are capable of inducing CYP1A expression as not all PAHs are equally capable of inducing CYP1A expression (Stagg et al., 2000). Analysis of additional biomarkers would further our knowledge as to the effects of chemical exposure on different fish populations (Eufemia et al., 1997). Additional studies, including the capture and subsequent breeding and crossbreeding between sites of bottom‐feeding species would strengthen discernment between adaptation and use of CYP1A for site assessment (Nacci et al., 1999). Finally, it would be beneficial to gather larger sample sizes of bottom‐feeding species specifically to increase confidence when drawing conclusions as differences in biomarker expression can exist between species as well (van den Hurk et al., 2017). It was interesting to collect species from different levels of the water column, and this could strengthen the relationship between CYP1A expression and bottom‐dwelling species versus nonbottom dwelling species if they exhibit less CYP1A expression in comparison.

It is important to note that since this study was conducted, various remedial efforts for these water sources have taken place. At Tifft Nature Preserve, there are ongoing restoration efforts based on a plan proposed in 2019 (Habitat Restoration Plan Niagara River Area of Concern, 2019). Clean‐up efforts at Genesee River are being organized under a voluntary clean‐up program (NYDEC). In an article posted last year, Monroe County officials stated that remediation of the Genesee River has progressed nearly to completion, but are still underway (Orr, 2020). The remaining aquatic sites explored in this study (Rushford Lake, Love Canal, Cuba Lake, Hanging Bog, and Moss Lake) have not had any recent remediation efforts (NYDEC).

## CONFLICT OF INTEREST

None declared.

## AUTHOR CONTRIBUTIONS

**Rebecca Williams:** Conceptualization (lead); Data curation (supporting); Formal analysis (lead); Funding acquisition (lead); Investigation (lead); Methodology (lead); Project administration (lead); Resources (lead); Software (lead); Supervision (lead); Validation (lead); Visualization (lead); Writing – original draft (supporting); Writing – review & editing (lead). **Theresa Taggart:** Conceptualization (equal); Data curation (equal); Formal analysis (equal); Funding acquisition (supporting); Investigation (equal); Methodology (equal); Project administration (equal); Resources (supporting); Software (supporting); Supervision (supporting); Validation (supporting); Visualization (supporting); Writing – original draft (equal); Writing – review & editing (equal). **Kayla Ganger:** Conceptualization (supporting); Data curation (equal); Formal analysis (equal); Funding acquisition (supporting); Investigation (equal); Methodology (equal); Project administration (supporting); Resources (supporting); Software (supporting); Supervision (supporting); Validation (supporting); Visualization (supporting); Writing – original draft (equal); Writing – review & editing (equal). **Seema Johnson:** Conceptualization (supporting); Data curation (equal); Formal analysis (equal); Funding acquisition (supporting); Investigation (equal); Methodology (equal); Project administration (supporting); Resources (supporting); Software (supporting); Supervision (supporting); Validation (supporting); Visualization (supporting); Writing – original draft (equal); Writing – review & editing (supporting). **Teri Koetsier:** Conceptualization (supporting); Data curation (equal); Formal analysis (equal); Funding acquisition (supporting); Investigation (equal); Methodology (equal); Project administration (supporting); Resources (supporting); Software (supporting); Supervision (supporting); Validation (supporting); Visualization (supporting); Writing – original draft (equal); Writing – review & editing (supporting). **Amber Dinchman:** Conceptualization (supporting); Data curation (supporting); Formal analysis (supporting); Funding acquisition (supporting); Investigation (supporting); Methodology (supporting); Project administration (supporting); Resources (supporting); Software (supporting); Supervision (supporting); Validation (supporting); Visualization (equal); Writing – original draft (supporting); Writing – review & editing (equal).

## Data Availability

The data that support the findings of this study are available at Dryad (DOI): https://doi.org/10.5061/dryad.7h44j0ztt.
